# Supplementary material for: Humorous peer play and social understanding in childhood
Source: Commun Psychol. 2025 May 12;3:76. doi: 10.1038/s44271-025-00252-3 (PMC12069696; doi:10.1038/s44271-025-00252-3)
Supplement: Supplementary file 2 — Supplemental Information [file 44271_2025_252_MOESM2_ESM.pdf]

Supplementary Notes

Deviation from Pre-registered Analyses

Our predictions regarding associations between children's total humour production and social understanding (amongst other predictions not addressed in this paper) were pre-registered https://osf.io/6cpvw (date of pre-registration 10/12/2022). In our original pre-registered analysis, we planned to account for partner effects by adjusting for nonindepedence using cluster-robust standard errors[1]. However, this approach would not enable us to evaluate the extent to which children's humour production is explained by partner effects[2]. The importance of this is underscored by recent papers indicating the overwhelming partner effects on children's observed play behaviours[3,4]. We therefore adopted a multi-level modelling approach (MLM) to evaluate partner effects and test direct associations between child factors and total humour production observed in the play interaction. This approach also enabled child-level variables and group-level variables to be modelled. In our study, children were observed in dyads and triads. MLM provides the advantage of accounting for this group-level variable in our model that would not have been possible otherwise.

Pre-registered Analysis

However, below we report the findings from the pre-registered analytical approach. In accordance with our pre-registered hypothesis, we detected a significant association between children's performance on the Triangles task and their total humour production in peer play (Table 3 in the main paper). This was followed up by examining this association while accounting for child age, gender, and receptive vocabulary, as these variables were associated children's total humour production or children's performance on the Triangles task (see Table 3 of main paper). The results from this regression analysis are presented in Supplementary Table 1. In accordance with the analyses presented in the main paper, only children's performance on the triangles task was significantly associated with children's total production of humour during peer play.

Supplementary Table 1. *Standardised model results for predictors of observed humour production during peer play.*

	Total humour production in peer play		
	Coefficient (SE)	95% CI	*p* value
Child age	-.016 (.124)	-.015 to .013	.895
Child gender	-.178 (.091)	-.309 to .003	.052
Receptive vocabulary	.068 (.113)	-.004 to .007	.547
Triangles ToM	.219 (.090)	.035 to .312	.015
R^2	.093		.076

Note. $N = 113$. Cluster-robust standard errors were used in the model to adjust for clustering of dyads and triads in peer observation (57 clusters).

Supplementary References

1 McNeish, D., Stapleton, L. M. & Silverman, R. D. On the unnecessary ubiquity of hierarchical linear modeling. *Psychological methods* **22**, 114 (2017). https://doi.org/10.1037/met0000078

2 Kenny, D. A., Kashy, D. A. & Cook, W. L. *Dyadic data analysis*. (Guilford Publications, 2020).

3 Goodacre, E. J., Fink, E., Ramchandani, P. & Gibson, J. L. Building connections through play: Influences on children's connected talk with peers. *British Journal of Developmental Psychology* **41**, 203-226 (2023). https://doi.org/10.1111/bjdp.12443

4 Gibson, J. L., Fink, E., Torres, P. E., Browne, W. V. & Mareva, S. Making sense of social pretense: The effect of the dyad, sex, and language ability in a large observational study of children's behaviors in a social pretend play context. *Social Development* **29**, 526-543 (2020). https://doi.org/10.1111/sode.12420
